# Concomitant oral intake of purified clinoptilolite tuff (G-PUR) reduces enteral lead uptake in healthy humans

**DOI:** 10.1038/s41598-021-94245-x

**Published:** 2021-07-20

**Authors:** Karolina Samekova, Christa Firbas, Johanna Irrgeher, Christine Opper, Thomas Prohaska, Anika Retzmann, Cornelius Tschegg, Claudia Meisslitzer, Anastassiya Tchaikovsky, Ghazaleh Gouya, Michael Freissmuth, Michael Wolzt

**Affiliations:** 1 Department of Clinical Pharmacology, Medical University of Vienna, Währinger Gürtel 18-20, 1090 Vienna, Austria; 2 Department of General, Analytical and Physical Chemistry, Montanuniversität Leoben, Franz-Josef-Straße 18, 8700 Leoben, Austria; 3 Glock Health, Science and Research GmbH, Hausfeldstraße 17, 2232 Deutsch-Wagram, Austria; 4 Gouya Insights, Elisabethstrasse 22/12, 1010 Vienna, Austria; 5 Institute of Pharmacology and the Gaston H. Glock Research Laboratories for Exploratory Drug Development, Center of Physiology and Pharmacology, Medical University of Vienna, Währingerstrasse 13a, Vienna, Austria

**Keywords:** Disease prevention, Public health, Therapeutics, Methods of toxicology studies, Medical toxicology

## Abstract

Lead exposure can cause substantial organ damage. Enteral lead absorption may be reduced by concomitant intake of clinoptilolite tuff, a zeolite from natural sources. This study aimed to assess the effect of purified clinoptilolite tuff (G-PUR) on enteral lead uptake in adults using stable lead isotope ^204^Pb as a tracer. In this randomized, placebo-controlled, double-blind, parallel-group study, 42 healthy participants were randomized to receive oral G-PUR 2.0 g, 2 * 2.0 g, or placebo, together with 2.5 µg of ^204^Pb in water. The enrichment of ^204^Pb caused by the tracer in blood and urine was measured by mass spectrometry. G-PUR was well tolerated. The mean maximum ^204^Pb enrichment of 0.505% of total blood lead was significantly higher (p < 0.0001) in the placebo group compared to G-PUR 2.0 g (0.073%) or G-PUR 2 * 2.0 g (0.057%) group. Normalized ^204^Pb AUC_0-192_ was 86.5, 11.9, and 8.5% * h without and with G-PUR 2.0 g, and G-PUR 2 * 2.0 g, respectively (p < 0.0001 vs. placebo). This smaller ^204^Pb exposure was paralleled by a reduced urinary excretion in subjects receiving G-PUR. Concomitant oral intake of purified clinoptilolite tuff reduced enteral uptake of ^204^Pb in healthy humans by approximately 90%. The reduced bioavailability is demonstrable by a decrease of ^204^Pb tracer enrichment in blood and urine.

**Trial registration**: clinicaltrials.gov identifier: NCT04138693, registered 24/10/2019.

## Introduction

Lead (Pb) is a natural toxic element ubiquitous in the environment^1^. Lead is released into the air and water during combustion of coal, oil, and waste; mining, through manufacture and application of Pb containing pesticides or Pb containing products like pigments, batteries or electrical shielding^2^. Exposure to lead happens typically through inhalation and ingestion of contaminated matrices such as soil, food or drinking water. Exposure to lead and other environmental contaminants is an important public health issue^2,3^. According to the European Food Safety Authority, there is no safe level of lead intake, because even trace amounts of lead may affect human health. Hence the PTWI (Provisional Tolerable Weekly Intake) of 25 µg/kg body weight was withdrawn in 2010^4^.

Acute and chronic lead toxicity affects almost every organ system in the human body. The nervous system is the most sensitive target of lead exposure^5^. Other typical symptoms are anaemia, renal failure and hypertension^2^. Weeks to months of chronic lead exposure are usually required to elicit manifestations of toxicity; however, acute signs and symptoms also occur from short-time intense exposures^6^.

After oral ingestion, lead is absorbed primarily in the upper small intestine^7^, which depends on a variety of factors, including the amount of calcium, phosphorus, zinc, iron, fat, protein, and vitamin D present in the intestines. Uptake is also influenced by the physical and chemical form and the amount of lead administered and the fasting status^8^. Mineralized tissues act as a sink for lead. Tight binding of lead to hydroxyapatite results in continuous accumulation and a very long half-life of lead (20–30 years), which is determined by bone turnover. Hence, in adults bones (and teeth) contain around 94% of total lead body burden^9^. The fraction of lead, which circulates in blood, is eliminated with a half-life of approximately 28–40 days via urine and faeces^10^.

Clinoptilolite is a natural zeolite, which binds heavy metals with high affinity, but is not absorbed during gastrointestinal passage. This capacity has been demonstrated in vitro and in animal studies^11–16^. Thus, oral administration of clinoptilolite may reduce the bioavailability of ingested lead in humans. We tested this hypothesis by concomitant administration of traces of the natural isotope ^204^Pb with a purified clinoptilolite preparation (GHC1, G-PUR) in water. This approach allowed for monitoring of changes in lead isotope pattern in blood and urine and avoided a major additional lead intake. Two different doses of G-PUR were applied to study the amount of purified clinoptilolite required for adsorption of ^204^Pb tracer in the gastrointestinal tract.

## Results

Subject characteristics at baseline are summarized in Table 1. Pharmacokinetic data of ^204^Pb are provided in Table 2. A substantial variability between subjects was noted for blood total Pb concentrations, which were lower in the G-PUR 2.0 g dose group (Table 3). However, blood total Pb concentrations were unchanged within groups during the observation period.

**Table 1:** Subject characteristics by treatment group at baseline.

	Placebo (*n* = 14)	G-PUR 2.0 g (*n* = 14)	G-PUR 2 * 2.0 g (*n* = 14)
Age (years)	27 ± 5	27 ± 4	23 ± 2
Sex (m/f)	7/7	7/7	7/7
Body mass index (kg/m^2^)	21.5 ± 1.5	22.7 ± 2.3	22.6 ± 2.3
Haematocrit (%)	40.2 ± 2.7	41.7 ± 3.3	41.0 ± 2.2
Whole blood lead (μg/l)	16.2 ± 7.7	14.1 ± 10.0	14.8 ± 11.6
Plasma ferritin (µg/l)	75.3 ± 64.5	80.8 ± 52.3	82.0 ± 56.9
Plasma creatinine (mg/dl)	0.8 ± 0.1	0.8 ± 0.1	0.8 ± 0.2

Absolute numbers and means ± *SD* are indicated.

**Table 2:** ^204^Pb pharmacokinetic parameters after single oral intake of 2.5 µg ^204^Pb in water by treatment groups.

	Placebo (*n* = 14)	G-PUR 2.0 g (*n* = 14)	G-PUR 2 * 2.0 g (*n* = 14)
*C*_max_ (%)	0.505 ± 0.281	0.073 ± 0.043	0.057 ± 0.022
*t*_max_ (h)	99 ± 72	94 ± 76	77 ± 63
AUC_0-192_ (% * h)	86.4 ± 49.4	11.9 ± 7.8	8.5 ± 3.1

^204^Pb enrichment is expressed as molar fraction (%) of total blood lead, normalized for hematocrit and body mass index. Data are means ± *SD*.

**Table 3:** Whole blood Pb concentrations by treatment groups over time.

	Placebo	Pb concentration (µg/l)	
		G-PUR 2.0 g	G-PUR 2 * 2.0 g
0 h	19.0 ± 12.5	13.9 ± 4.3	17.3 ± 13.2
4 h	18.9 ± 12.5	13.3 ± 3.8	15.6 ± 10.2
24 h	19.6 ± 12.5	13.4 ± 4.0	15.1 ± 10.2
48 h	19.3 ± 12.4	13.6 ± 4.2	15.4 ± 10.5
192 h	19.6 ± 12.9	14.5 ± 6.0	15.1 ± 10.0

Data are means ± *SD* (*n* = 14 per group).

**Figure 1:**
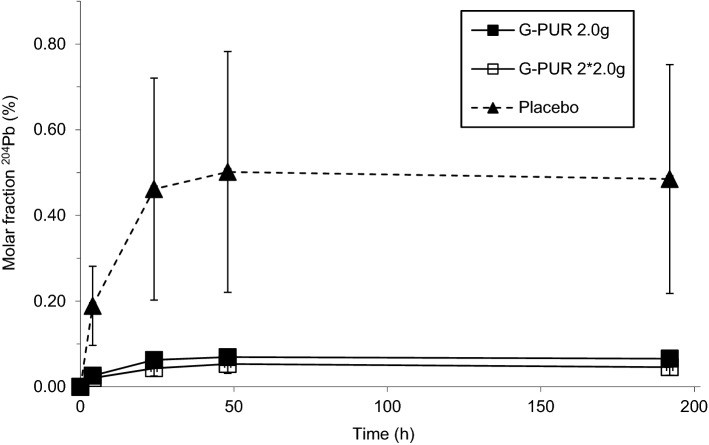
Molar fraction of ^204^Pb of total blood lead (% of individual total Pb at each time point, normalized for haematocrit and body mass index) after single oral intake of 2.5 µg ^204^Pb with purified clinoptilolite (G-PUR) at 2.0 g, 2 * 2.0 g, or placebo. Means ± *SD* are indicated, *n* = 14 per group.

After single oral dosing, ^204^Pb enrichment in blood increased to a mean C_max_ of 0.505% of total Pb in the absence of G-PUR intake. ^204^Pb enrichment was slow and reached a plateau between 24 and 48 h, which was maintained until day 6 after dosing (Fig. 1). Concomitant ingestion of G-PUR 2.0 g or of G-PUR 2 * 2.0 g resulted in a significantly smaller ^204^Pb-tracer enrichment in blood compared to placebo (all: p < 0.0001, Fig. 1), with a mean C_max_ of ^204^Pb of 0.073% or 0.057% of total Pb, respectively. This reduction in gastrointestinal ^204^Pb absorption in participants receiving G-PUR also resulted in a substantially lower mean AUC_0-192_ of ^204^Pb enrichment in blood compared to placebo (all: p < 0.0001; Table 2). Based on the comparison of AUC_0-192_-values, administration of 2.0 g and 2 * 2.0 g G-PUR reduced ^204^Pb absorption on average by 86% and 90%, respectively. The t_max_ of ^204^Pb-tracer in blood was not affected by co-administration of purified clinoptilolite (Fig. 1, Table 2).

Consistent with lower lead bioavailability, ^204^Pb excretion in 24-h urine was also substantially smaller with G-PUR compared to placebo. Mean ^204^Pb mass content per g creatinine in urine was 14.1 ± 9.3 ng g^−1^ after placebo, and 2.8 ± 3.8 ng g^−1^ and 3.7 ± 3.0 ng g^−1^ after G-PUR 2.0 g and G-PUR 2 * 2.0 g, respectively.

G-PUR was well-tolerated and no serious adverse events were reported. Thirty-three adverse events were recorded, 16 of which were classified as moderate in intensity (Table 4). The most frequent adverse event was headache, which was reported by 5, 3, and 6 participants receiving placebo, G-PUR 2.0 g, and G-PUR 2 * 2.0 g, respectively.

**Table 4:** Adverse events by treatment group, severity and system organ class category.

	Placebo (*n* = 14)	G-PUR 2.0 g (*n* = 14)	G-PUR 2 * 2.0 g (*n* = 14)
Total	9	7	17
**Severity**			
Mild	3	2	12
Moderate	6	5	5
**System organ class**			
Gastrointestinal disorders	0	1	3
Infections and infestations	4	1	1
Nervous system disorder	5	3	6
Reproductive system and breast disorders	0	0	1
Respiratory, thoracic and mediastinal disorders	0	1	2
Skin and subcutaneous tissue disorders	0	1	2
Vascular disorders	0	0	1
Fatigue	0	0	1

## Discussion

In the present study, we demonstrated that G-PUR reduces the gastrointestinal absorption of ^204^Pb in fasting healthy humans. This was shown under standardized conditions with two different doses of G-PUR, which caused a reduction of ^204^Pb concentrations in whole blood to less than 15% of C_max_ seen with placebo. The purified clinoptilolite tuff preparation under study was therefore highly effective in our experimental model to prevent uptake of lead after a single gastrointestinal exposure of ^204^Pb as tracer.

The current controlled trial further suggests that the selected doses of G-PUR were at the upper end of the dose–response curve, i.e. close to saturation. The dose of 2 * 2.0 g G-PUR reduced uptake ^204^Pb—as estimated by C_max_ or AUC_0-192_—by some additional 3–4%, when compared to the level achieved the dose of 2.0 g. This is consistent with the law of mass action, which predicts only a very modest increase in adsorption of ^204^Pb G-PUR by doubling of the G-PUR dose, as binding approaches saturation. We stress, however, that the pharmacokinetic endpoints of the study were not powered to ascertain statistically significant differences between such small differences in ^204^Pb enrichment at different G-PUR doses. In this trial, we used a micro-dose of ^204^Pb (2.5 µg/250 ml), which met the recommended upper limit of lead concentration in drinking water. The binding capacity of clinoptilolite tuff is in the range of 0.8 mmol Pb/g clinoptilolite, i.e. 166 mg Pb/g^17^. Thus, while not formally proven for obvious ethical reasons, it is reasonable to assume that the dose range of purified clinoptilolite tuff, which we investigated in the present study, also suffices to prevent the intestinal absorption of substantially larger amounts of lead. This assumption is justified by experiments in piglets, which were fed substantially larger amounts of lead (50 mg and 100 mg Pb⋅CH_3_COO/100 g total feed) for 5 weeks. In spite of these excessive amounts of lead, concomitant administration of clinoptilolite tuff reduced the tissue accumulation of lead by 50%^18^.

Natural clinoptilolite tuff is contaminated with various heavy metals and metalloids (e.g. As2O3). A purification procedure is therefore required to ascertain the removal of the contaminants and to provide a non-hazardous product for human use. The current purified clinoptilolite tuff preparation G-PUR fulfils this criterion. The mechanism by which G-PUR has abrogated ^204^Pb enrichment in blood is explained by the strong affinity of clinoptilolite tuff for heavy metals^14,19,20^. Direct adsorption of ^204^Pb after concomitant intake has presumably prevented transepithelial ^204^Pb uptake and transfer into the blood compartment. However, we did not collect faeces to confirm this sequestration of ^204^Pb in the gastrointestinal tract.

In the treatment of acute lead poisoning, chelation therapy plays a major role. Chelating agents such as dimercaprol or succimer pass into the bloodstream and bind lead. These lead-chelation complexes are then excreted via the urine^1^. However, chelation therapy requires repeated hospital stays and is accompanied by major side effects. Thus, it is only suitable for treating severe Pb poisoning^21^. In comparison, clinoptilolite tuff is an easy-to-use powder. Its administration requires mixing the powder with water and drinking the suspension. Due to the size of its grains of several micrometer (µm) and its chemical inertness, clinoptilolite tuff does not pass into the bloodstream. Its main mechanism of action is binding Pb from water and food in the gastrointestinal tract. The Pb loaded clinoptilolite tuff is then excreted via the feces. This prevents Pb absorption into the bloodstream, which is beneficial for mitigation of chronic low-level lead exposure^22^.

Of note, uptake of ^204^Pb was not delayed by concomitant purified clinoptilolite tuff ingestion as shown by similar t_max_ values between groups, despite the marked reduction in bioavailability caused by G-PUR. This study did not assess the mechanisms of lead uptake and blood sampling was applied only at a few time points.

However, the present data are also of interest to understand intestinal absorption of lead in people. Enteral uptake of lead is mediated by the divalent metal transporter 1 (DMT1/SLC11A2, solute carrier 11A2), a proton-coupled intestinal membrane transporter for ferrous iron^23^. Iron deficiency increases the expression of DMT1^24,25^. Accordingly, the bioavailability of lead is increased in iron deficiency^26,27^. However, lead levels in blood and iron status are not always inversely correlated^28^. Hence, an additional transport mechanism must exist, which supports intestinal absorption of lead. In fact, in Caco-2 cells, which are derived from a human colonic adenocarcinoma and which express DCT1^29^, lead uptake is reduced but not abolished by knock-down of DCT1^30^. Similarly, murine ileum transports substantial amounts of lead by a mechanism that is independent of DCT1^31^. In the present study, we observed that the enrichment of ^204^Pb in whole blood rose slowly and was similar between 24 and 48 h after dosing (Fig. 1). Thus, the time profile of our pharmacokinetic data with a mean ^204^Pb enrichment t_max_ of > 96 h supports the concept that a considerable portion of enteral lead is not solely absorbed into blood by the proximal intestinal DMT1-mediated transport alone, but also in the more distal parts of the small intestine. Also, it is possible that G-PUR binds already directly to lead in the stomach, in particular when the interval between oral intake is short. To the best of our knowledge, our findings are the first to provide evidence for absorption from the distal parts of the human intestine. The use of ^204^Pb as a tracer for lead kinetics was introduced in the experiments by Rabinowitz et al.^10,32,33^. However, a substantially higher dosing regimen up to 200 µg ^204^Pb was administered in multiple doses. Hence, it was not possible to study the kinetics of absorption. In contrast, our study has employed a single dose of as little as 2.5 µg ^204^Pb. The refined analytical method enables micro-dosing of humans and exposure of lead well below limits of maximum daily intake.

There are several limitations of this study. Firstly, only healthy humans were eligible and the presence of gastrointestinal abnormalities or transit time may alter the findings. Secondly, ^204^Pb intake was under acute and fasting conditions, which is known to optimize lead absorption and therefore to increase the detection of potential differences in bioavailability caused by G-PUR.

No relevant or new risks of G-PUR were identified in the present healthy study population. This is in good agreement with the fact that this product and other zeolite preparations are already available on the market for several years and that no safety concerns have been reported. However, other signals may arise from patients after multiple dosing. A particular caution may be necessary to avoid a drug interaction with this compound, which could reduce oral bioavailability of other medicines when taken concomitantly.

In conclusion, this study demonstrates that oral purified clinoptilolite efficiently reduces enteral lead uptake in healthy humans. The clinical potential of G-PUR to mitigate the toxicity of acute or chronic enteral lead exposure should be confirmed in appropriately designed trials accordingly.

## Methods

This trial was conducted as a medical device clinical trial according to the regulatory provisions in Austria. Glock Health, Science and Research GmbH acted as sponsor of this single centre study. The study protocol was approved by the Ethics Committee of the Medical University of Vienna (no. 1285/2019) and the Austrian Federal Office for Safety in Health Care (BASG) and is registered at clinicaltrials.gov (NCT04138693). This study was conducted at the Department of Clinical Pharmacology at the Medical University of Vienna in accordance with the Declaration of Helsinki. All study participants provided written informed consent before any study-specific procedures were performed. The study was initiated on 28th of August 2019 and the clinical phase completed on the 12th of February 2020.

G-PUR is marketed in the United States as a dietary supplement. It is a purified clinoptilolite-tuff, prepared from a high-grade raw material, sourced from a mine in the eastern Slovak Republic^34,35^. The content of clinoptilolite in the purified product that was processed from the raw material is > 75%. Other mineral phases are cristobalite, feldspars and accessory biotite and quartz. The patented purification process is technically based on ion exchange mechanisms of the clinoptilolite mineral, micronisation and terminal heating, which results in the removal of all natural impurities and a homogeneous, very fine-grained particle size^36^. The production process is thoroughly quality assured meeting all required regulatory standards; the purified product has been evaluated by independent laboratories and conforms with the safety requirements for human consumption. The final product was called GHC1 in previous studies and is marketed under the name G-PUR^37^.

The primary objective was to assess the effect of G-PUR on enteral lead absorption in adults using a stable lead isotope as tracer (^204^Pb). Secondary objectives were to evaluate the safety and tolerability profile of G-PUR at two different doses, pharmacokinetic parameters in blood and the assessment the effect of G-PUR on urinary excretion of ^204^Pb.

The primary pharmacokinetic endpoint was the maximum enrichment of ^204^Pb in blood after intake of ^204^Pb-spiked water. The stable isotope ^204^Pb was selected as tracer in the present experiments. Its natural abundance is in the range of < 2% of total lead, which makes ^204^Pb a suitable candidate for lead absorption studies^10,32,33^. Enrichment of ^204^Pb was expressed as the molar fraction of total lead at each individual time point expressed as x (^204^Pb-tracer, %). ^204^Pb-tracer (%) was further normalized to haematocrit/body mass index obtained at baseline (t = 0) to account for physiological differences between subjects.

Secondary endpoints were (i) the area under curve of the normalized ^204^Pb-tracer (%) versus time curve up to 192 h (AUC_0-192_), (ii) the time to reach C_max_ (t_max_), (iii) the 24-h urinary excretion of tracer-^204^Pb per g creatinine, and (iv) safety, based on the frequency and severity of adverse events.

### Study procedures

The study followed a randomized, placebo controlled, double-blind, parallel-group design. Male or female healthy volunteers aged 18–45 years with a body mass index (BMI) of 19–27 kg/m^2^ for males and 17–25 kg/m^2^ for females were eligible for participation. Main inclusion criteria were whole blood lead concentration < 40 μg/l as measured by graphite furnace atomic absorption spectroscopy^38^, serum ferritin concentration within the sex-specific normal range and 1 month of stable eating habits. Key exclusion criteria were pregnancy or breastfeeding in women, regular use of medication or iron supplements in the previous 2 months, any relevant organ disease, especially gastrointestinal pathology, recent diarrhea, diabetes, symptomatic food allergies or aluminium and/or silicon hypersensitivity. Participants were excluded, if they showed any clinically relevant laboratory abnormalities at screening, or alcohol, nicotine or drug abuse.

Forty-two participants were studied in fasting state. Volunteers were randomized into one of three parallel groups, who received placebo drinking solution (2 * 100 ml water), 2.0 g G-PUR suspended in 100 ml water and placebo drinking solution of 100 ml water, or 2 * 2.0 g G-PUR suspended in 100 ml water each, together with 250 ml still mineral water containing 2.5 μg of ^204^Pb. G-PUR is tasteless. A preparation of 3 opaque drinking bottles per subject was done by an unblinded study nurse to maintain masked conditions for the blinded participants and investigators. All bottles had to be ingested in predefined order (placebo/G-PUR—^204^Pb spiked water—placebo/G-PUR) within 5 min. Special protective precautions were undertaken to avoid cross-contamination with ^204^Pb spiked solution.

204Pb was purchased at BuyIsotope (Solna, Stockholm/Sweden) in metal form with an isotopic enrichment of 99.94% ^204^Pb. 11.74 mg of this metal was dissolved in 5.00 ml of nitric acid (w = 15%; p.a./Ph. Eur., VWR) and diluted with 18.2 MΩ.cm water to a final concentration of 2681 ng/ml of ^204^Pb (at a pH of 2.5 and a HNO_3_ concentration of 201.2 mg NO_3_^-^/l). This stock solution was diluted to a final ^204^Pb concentration of 10.0 µg/l (which corresponds to the Austrian legal limit for Pb in tap water^39^) one hour before administration to avoid stability issues.

Venous blood samples were taken at baseline (t = 0) and at scheduled time points. Blood was drawn into lithium heparin containing tubes, processed if applicable, and stored at − 20 °C until analysis.

The kinetics of oral ^204^Pb tracers in humans are not appropriately characterized. Hence, we used a staged approach for adjusting pharmacokinetic blood sampling. For the first six subjects, blood samples were collected at baseline, and at 4 h, 8 h, 12 h, 24 h, 48 h and 192 h after ^204^Pb administration. Based on the preliminary results obtained, which were masked for the investigator, blood sampling time points were adapted for the 36 subsequent subjects according to the study protocol: sampling at 8 h and 12 h was omitted, because these time points were dispensable for defining C_max_. Urine was collected at baseline and during the first 24 h after ^204^Pb administration.

For analysis, an aliquot of whole blood (0.5 ml) which was not frozen was pre-digested on site using 4 ml double subboiled HNO_3_ (w = 65%). After cooled transfer to the analytical laboratory, 2 ml HNO_3_ (w = 65%) and 0.5 ml H_2_O_2_ (w = 30%) were added to the pre-digested blood samples and allowed to incubate for > 10 min prior to mixing in closed tubes to avoid strong gassing of the samples during reaction before transferring the solution into microwave vessels.

The digestion of blood samples was performed by microwave assisted digestion (Multiwave PRO, Anton Paar GmbH, Graz, Austria) using rotor type 48MF50. Prior to digestion of the samples, the microwave vessels were cleaned twice with double subboiled HNO_3_ (6 ml, w = 65%). The microwave program was ramped up to 1400 W for 15 min and held at power for 20 min before cooling for 15 min. Digested blood samples were quantitatively transferred to 10 ml test tubes and stored at 4 °C until further processing.

Total lead and ^204^Pb was quantified in the liquid samples using a quadrupole inductively coupled plasma mass spectrometer (ICP-QMS, NexION 2000b, PerkinElmer, Ontario, Canada) coupled to an ESI SC-2 DX FAST autosampler (ESI, Omaha, Nebraska, US)^40,41^. The limits of quantification were 0.09 ng Pb/g blood and 0.001 ng ^204^Pb/g blood, respectively.

### Sample size and randomization

Sample size calculation was based on the assumption that the bioavailability of ^204^Pb was 39% and that C_max_ of ^204^Pb was reduced by at least 38% after intake of G-PUR when compared to placebo. A 2-sided confidence level of α = 2.5% (adjustment for multiple testing) and a power of 80% were used (nQuery Version 4.0.0.0), including a 15% drop out rate. Based on these calculations, a total sample size of 42 (14 per group) subjects was randomized in a 1:1:1 fashion. To ensure equal randomization across sexes, a separate randomization list was generated for male and female subjects and block randomization with sex as a block factor was used. Subjects were randomized in fixed blocks of three to ensure that the balance between the treatment groups was maintained.

### Statistical analysis

The parameters of ^204^Pb kinetics were assessed using a non-compartmental model. The primary endpoint (C_max_ of ^204^Pb, normalized for individual total Pb concentrations, haematocrit and BMI) was evaluated for statistical differences between groups based on the full analysis set. The two treatment groups were tested against the placebo group. The Bonferroni correction (significance level α/2 = 2.5% two-sided) was used to adjust for multiple testing. The two-sample t-test was used for each of the hypotheses, and descriptive statistics of the main parameters was performed. The parameters of the secondary endpoints were analysed using appropriate statistical tests. These tests were performed for explorative purposes only and included t-tests, analysis of variances and analysis of covariances. In addition, appropriate methods for adjusting for baseline imbalances were applied. Safety parameters were evaluated by descriptive statistics. The statistical analysis was done using the software package SPSS Version 26.

## Data Availability

The data that support the findings of this study are available from the corresponding author, upon reasonable request.
